# Comparison and Characterization of the Structure and Physicochemical Properties of Three Citrus Fibers: Effect of Ball Milling Treatment

**DOI:** 10.3390/foods11172665

**Published:** 2022-09-01

**Authors:** Zhanmei Jiang, Minghan Zhang, Yuxuan Huang, Chenglong Ma, Sinan Mu, Hongyu Li, Xianqi Liu, Yue Ma, Yue Liu, Juncai Hou

**Affiliations:** Key Laboratory of Dairy Science, Ministry of Education, College of Food Science, Northeast Agricultural University, Harbin 150030, China

**Keywords:** citrus fiber, ball milling, functional properties, physicochemical properties

## Abstract

Effects of ball milling (BM) on the structure and physicochemical properties of three types of citrus fibers were investigated. With the extension of the grinding time, the particle size of citrus fibers significantly decreased. Fourier transform infrared spectroscopy (FTIR) showed that the three citrus fibers had similar chemical groups, and more -OH and phenolic acid groups were exposed after BM, and pectin and lignin were not degraded. Scanning electron microscope (SEM) results showed that the appearance of particles changed from spherical to fragmented, irregular shapes. The water holding capacity (WHC), oil holding capacity (OHC), and water swelling capacity (WSC) of citrus fibers LM, JK, and FS reached the maximum value after BM of 2 h (increasing by 18.5%), 4 h (increasing by 46.1%), and 10 h (increasing by 38.3%), respectively. After 10 h BM, citrus fibers FS and JK had the highest adsorption capacity of cholesterol and sodium cholate, increasing by 48.3% and 48.6%, respectively. This indicates that BM transforms the spatial structure of citrus fibers and improves their physicochemical properties.

## 1. Introduction

Citrus is one of the most cultivated fruit crops in the world, with annual production of more than 124 million tons [1]. Worldwide, the quantity of citrus by-products was estimated to exceed 1.5 million tons. The global citrus processing industry generates more than 110 million tons of citrus waste [2]. Citrus waste, including citrus peels, pulp residues, and seeds, accounts for 40–60% of the total fruit mass. In the process of citrus fruit processing, citrus waste is a burden to the environment without further treatment. In order to maximize the utilization of citrus waste, citrus pomace is widely applied in the pectin extraction industry [3].

Citrus fiber is the dietary fiber extracted from citrus waste. It consists mainly of pectin extracted from insoluble dietary fiber (IDF) [4]. Studies have shown that citrus fiber has higher total dietary fiber (TDF) and better physicochemical properties than cereal fiber, such as water holding capacity (WHC) and water swelling capacity (WSC). In addition, research has found that citrus fiber also has excellent physiological functional properties. Citrus fiber is composed of IDF, SDF, pectin, and fructooligosaccharides. These components selectively stimulate the growth and activity of the intestinal microbiota, especially lactobacillus and bifidobacterium. Therefore, the intake of dietary fiber has a positive impact on the development of gut microbes [5,6]. The water-soluble dietary fiber in citrus fiber can adsorb cholesterol and reduce cholesterol in the body [7]. The composition and content of dietary fiber are different among various kinds of citrus fibers, and are related to the source and extraction method of dietary fiber.

The physicochemical properties of citrus fiber have been studied extensively. Many hydroxyl groups are present in the cellulose side chain in citrus fiber, and can combine with H_2_O to generate more stable hydrogen bonds, leading to good WHC. Due to its loose surface structure and small space barrier, citrus fiber can contain more water and have WSC [8]. The adjacent cellulose chains form a stable fibrous structure through hydrogen bonds, which makes cellulose in citrus fiber have oil holding capacity (OHC) [9]. It is widely believed that dietary fiber (DF) can improve the function properties of foods; for example, incorporation of dietary fiber in some foods can improve the viscosity of food and the ability to form gels [8]. The authors of [10] added citrus fiber to ice cream and found that citrus fiber can improve the sensory properties and non-fusibility of ice cream. The authors of [11] added citrus fiber to gluten-free corn breads, which showed that citrus fiber can improve the WHC of bread, and the incorporation of citrus fiber reduced the firmness of bread. Gedikoğlu and Clarke reported that all ground beef meatballs with citrus fiber were lower in saturated fatty acids and higher in dietary fiber compared to the control samples [12]. The authors of [13] added citrus fiber to sausage and found it can also decrease residual nitrite levels.

A large amount of research has showed that the modification technology of citrus fiber can enhance the physicochemical properties of dietary fiber. The modification methods are divided into physical and chemical methods, of which the physical method is the most common application. It includes ultrasonic treatment, high pressure homogenization treatment, ultrafine grinding treatment, and extrusion treatment [14]. Among these, ball milling (BM) is a kind of physical modification method which has a low cost and is friendly to the environment [15].

BM treatment can effectively decrease the particle size, and change the surface area and physicochemical properties [16]. For instance, the authors of [17] showed that BM significantly increased the WHC of the insoluble fiber-rich fraction and cellulose. Furthermore, the authors of [18] found that rice bran dietary fiber can remarkably enhance WHC, WSC, and nitrite ion adsorption capacity after ultrafine grinding. The authors of [19] also reported that the surface of citrus dietary fiber was smoother and its fluidity was higher after ultrafine grinding, and the cation exchange ability and metal cation binding ability were improved.

Although BM treatment has been applied for modification of citrus dietary fiber, it has been not expounded whether there are remarkable differences in the characteristics of commercial citrus fibers of various sources after BM treatment. Thus, the main innovation of this study is to highlight the modification effect of ball milling on citrus fibers from different sources. A comparison and characterization of the structure and physicochemical properties of three commercial citrus fibers after ultrafine grinding treatment were undertaken in this study. Additionally, the characteristic differences of three commercial citrus fibers after BM treatment were compared and analyzed through detection of their soluble dietary fibers (SDFs), IDF and TDF content, particle size, WHC, OHC, WSC, cholesterol adsorption capacity (CAC), sodium cholate adsorption capacity, Fourier transform infrared spectroscopy (FTIR), and scanning electron microscopy (SEM). After comparing the structure and physicochemical properties of the three citrus fibers, we selected an optimal citrus fiber for further in-depth research. This investigation provides basic data support for modification of commercial citrus fibers and broadens their application in the food industry.

## 2. Materials and Methods

### 2.1. Materials

The citrus fiber FS (5.7% protein, 1.12% fat, 5.36% moisture, 3.75% ash) was acquired from Fiberstar Biotechnology Co., Ltd., River Falls, WI 54022, USA. The citrus fiber LM (5.91% moisture, 5.04% protein, 3.75% ash, 0.79% fat) was acquired from Laimeng Biotechnology Co., Ltd., Guangdong, China. The citrus fiber JK (5.94% moisture, 5.21% protein, 3.13% ash, 0.87% fat) was acquired from JinKangjun Biotechnology Co., Ltd., Guangdong, China. Cholesterol, furfural, and sodium cholate was purchased from Solarbio Science and Technology., Ltd., Beijing, China. Soybean oil was purchased from Jiusan Grain and Oil Industry Group Co., Ltd., Heilongjiang, China.

### 2.2. Preparation of Modified Citrus Fiber by BM

Three citrus fiber powders were placed in the jar of a QM-ISP2 planetary ball mill (Nanjing University Instrument Factory, Nanjing, China). Mill balls (25 g) were added to the citrus fiber powders at the ratio of 1:8. The mill equipment was rotated at a speed of 560 rpm. BM was carried out for 2, 4, 6, 8, and 10 h. After BM, samples were stored for further use.

### 2.3. Determination of Citrus Fiber Components

Moisture was determined by drying a 10 g sample at 130 ± 3 °C for 3 h (AOAC, 1997). Ash was produced at 550 °C for 2 h (AOAC, 2000). Protein was analyzed according to the Kjeldahl method, and the conversion factor was 6.25 (AOAC method 2001.11). Fat was calculated by weight loss after a 6-cycle extraction with petroleum ether (AOAC, 2000).

### 2.4. Determination of SDF, IDF and TDF Content

TDF, IDF, and SDF contents were measured by the AOAC method 991.43 (AOAC, 2000). TDF was determined as follows: 225 mL ethanol was added to the sample and precipitated for 1 h. Residue was washed with 78% ethanol, 95% ethanol, acetone, and dried at 105 ℃. IDF and SDF were determined as follows: Enzyme digestate was filtered into residue and filtrate. Residue was washed and dried at 105 ℃ to obtain IDF. A quantity of 80 g filtrate and washing water was mixed with 320 mL ethanol and precipitated for 1 h. The residue was washed with 78% ethanol, 95% ethanol, and acetone, and dried at 105 ℃ to obtain SDF.

### 2.5. Particle Size Analysis

The particle size distribution and median particle size of three citrus fibers were measured according to the authors of [20] using a laser diffraction particle analyzer (HYL-1076, Haoyu Technology Co., Ltd., Liaoning, China). The sample groups and control groups were diluted with deionized water to a concentration of 0.5 mg/mL. Further, Ultrasonic-assisted dispersion was used before measurement. The refractive index of the sample was set at 1.460, and the absorption rate was set at 0.1.

### 2.6. Determination of WHC

WHC of the samples was analyzed according to the method described by [21] with a small modification. Initially, each sample (1.0000 g) was mixed with distilled water (20 mL) for 24 h, and the mixture was centrifuged (3000× *g*, 15 min). The calculation formula of WHC is as follows:

$$\mathrm{WHC}\;(g/g)=\frac{M_{2\;}-{\;M}_{1}}{M_{0}}$$

where M_2_ is the weight of the sample in the centrifuge tube after supernatant removal, M_1_ is the weight of the centrifuge tube, and M_0_ is the weight of the sample.

### 2.7. Determination of OHC

OHC was determined using a modified method from [22]. Each sample (0.5000 g) was mixed with 5 mL of oil and kept for 24 h, and then centrifuged (3000× *g*, 15 min).

The calculation formula of OHC is as follows:

$$\mathrm{OHC}\;(g/g)=\frac{M_{2}-{\;M}_{1}}{M_{0}}$$

where M_2_ is the weight of the centrifuge tube and oil-contained sample, M_1_ is the weight of the centrifuge tube, and M_0_ is the weight of the sample.

### 2.8. Determination of WSC

WSC was determined using a method described by [23]. Initially, 0.2 g of citrus fiber samples was weighted. Subsequently, distilled water (10 mL) was added to this vessel, and it was stored for 24 h at room temperature.

The calculation formula of WSC is as follows:

$$\mathrm{WSC}\;(\mathrm{mL}/g)=\frac{V_{2}-{\;V}_{1}}{M_{0}}$$

where V_2_ is the volume of the hydrated sample, V_1_ is the volume of the dried sample, and M_0_ is the weight of the dried sample.

### 2.9. Determination of CAC

CAC was determined using a modified method [24].

Fresh egg yolk was diluted 10-fold with distilled water. Mixtures of samples (1.0 g) and diluted yolk (25 mL) at pH 2.0 and 7.0, respectively, were shaken at 37 °C (120 rpm, 2 h). Then, it was centrifuged (2000 rpm, 15 min). The supernatant (1 mL) was diluted with the quintupling volume of glacial acetic acid. The supernatant dilution (400 µL), phthaladehyde color reagent (1.5 mL), and sulfuric acid (1 mL) were mixed. The absorbance was recorded at 550 nm.

The standard curve was drawn with the cholesterol standard solution (0.025, 0.050, 0.075, 0.100, and 0.125 mg/mL) as the abscissa and the absorbance value as the ordinate (the regression equation was y = 0.0159x + 0.0028, R^2^ = 0.9959). CAC was calculated using the standard curve.

### 2.10. Determination of Adsorption Capacity of Sodium Cholate

The adsorption capacity of sodium cholate was determined using a modified method [25].

Briefly, 1.00 g sample and 0.1 g sodium cholate were dissolved with 50 mL of 0.15 mol/mL NaCl (pH 7.0), and shaken (120 rpm, 2 h) at 37 °C. Subsequently, this mixed solution was centrifuged (2000 rpm, 15 min) and supernatant was collected. Further, three reagents of supernatant (1 mL), concentrated sulfuric acid with a concentration of 45% (6 mL), and furfural at a concentration of 0.3% (1 mL) were mixed well and placed in a water bath (65 °C, 30 min). After cooling, the absorbance was measured at 620 nm. The standard curve was drawn with sodium cholate (0.025, 0.050, 0.075, 0.100, and 0.125 mg/mL) as the abscissa and the absorbance value as the ordinate (the regression equation was y = 0.5431x + 0.0221, R^2^ = 0.9980). The adsorption capacity of sodium cholate was calculated using the standard curve.

### 2.11. Fourier Transform Infrared Spectroscopy (FTIR)

FTIR was measured by a reported method [26]. The infrared spectrum was performed in a total reflection FTIR instrument (AVATA 360, Nicolet, Thermo Fisher Scientific, Madison, WI, USA). The dry sample powder was mixed with KBr (1:100, *v*/*v*), and the spectra were obtained between 400 and 4000 cm^−1^.

### 2.12. Scanning Electron Microscopy (SEM)

The micrograph was observed according to a method described by [27] using a SEM (EVO 18, ZEISS, Oberkochen, Germany) at 5.0 KV. Each micrograph of samples was taken at 500–2000× magnification.

### 2.13. Statistical Analysis

Statistical analyses were performed using an analysis of variance (ANOVA) procedure of the SPSS 25.0. Significant differences (*p* < 0.05) of means were determined by the Duncan test, and the figures were drawn with Origin 2019. All tests were carried out in triplicate and the results are presented as mean values ± standard deviation.

## 3. Results and Discussion

### 3.1. The Content of IDF, SDF and TDF

Effects of BM on IDF, SDF, and TDF contents of three citrus fibers are shown in Table 1. The TDF content of FS, JK, and LM was significantly different, and the contents of TDF and IDF in citrus fiber LM were the highest among the three citrus fiber products (*p* < 0.05). In addition, citrus fiber FS has the highest SDF content. The authors of [28] investigated the dietary fiber composition of five types of pummelo and showed that Shatianyu had the highest TDF content. The authors of [29] also demonstrated that citrus fibers from chemical industry sources had higher lignin and cellulose contents, whereas citrus fibers from food industry sources had higher hemicellulose and pectin contents. However, the author of [24] reported the chemical composition of five citrus fibers, and found that there were no remarkable distinctions in the contents of TDF, SDF and IDF in five citrus fibers. The above research results show that composition of citrus fiber from different sources was different.

The contents of TDF and IDF of three citrus fibers were significantly decreased after BM, whereas the contents of SDF were significantly increased (*p* < 0.05). Generally, the hydrogen bond between the molecules of IDF in citrus fiber was potentially broken during the process of BM, resulting in transfer of branch-chain celluloses into short-chain soluble amorphous celluloses [30]. Another possibility is that BM may destroy the cell structure of citrus pomace, thus releasing soluble polysaccharides in the cells, leading to the increase in SDF [31].

### 3.2. Particle Size Distribution

**Figure 1:**
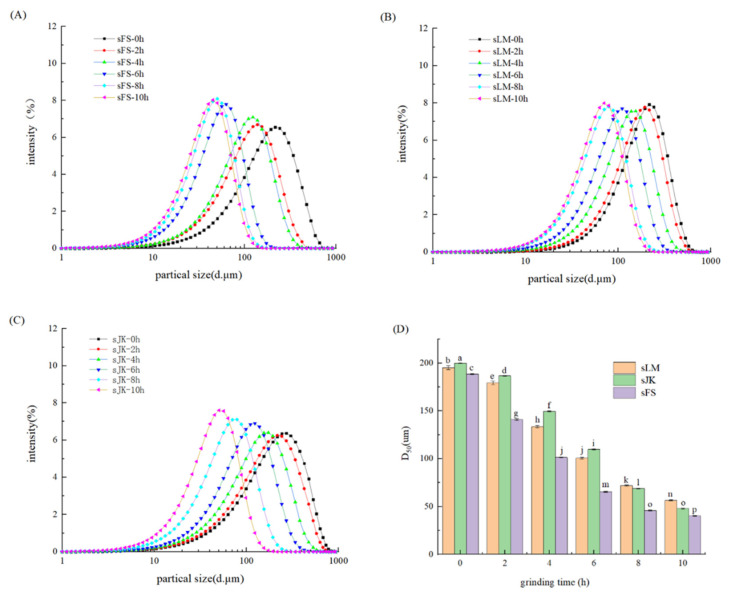
Effects of BM on particle size distribution of LM (**A**), JK (**B**), and FS (**C**). Average particle size of BM-treated citrus fibers (**D**). Error bars represent the standard deviation of the mean of triplicate experiments. Different letters (a–p) indicate significant difference (*p* < 0.05).

Particle size analysis is an important indicator for evaluating physicochemical properties [32,33,34]. Effects of BM on particle size distribution and median particle size (D_50_) of three citrus fibers are shown in Figure 1A–D. D_50_ is the median particle size and is commonly used to represent the average particle size of powders [35]. From Figure 1A–C, the particle size distribution of three kinds of citrus fibers was moved to the left and became narrower in the grinding time between 2–10 h. D_50_ of FS, LM, and JK decreased from 188.52 ± 0.83 μm, 195.31 ± 2.00 μm, and 200.07 ± 0.474 μm to 40.44 ± 0.31 μm, 56.65 ± 0.50 μm, and 47.85 ± 0.42 μm, respectively. From Figure 1D, the particle size of JK was the largest before modification, whereas the particle size of LM was the largest after 10 h of BM. In addition, it can be concluded that BM treatment has the greatest influence on the particle size of JK, which decreased by 152.22 μm. This may be due to the different sources of three citrus fibers. The authors of [17] found that BM (5 and 10 h) remarkably (*p* < 0.05) decreased the particle size of the carrot insoluble fiber-rich fraction to 58.4 μm (−55.8%) and 12.4 μm (−90.6%). A study by [36] showed that the D_50_ of citrus fibers gradually decreased with BM from 4 to 10 h under the same HPH pressure conditions (*p* < 0.05).

Furthermore, Figure 1D also indicates that D_50_ of three dietary fibers greatly declined (*p* < 0.05). This may be due to the grinding force generated by the impact between the balls during the grinding process, which makes the particle size of the three kinds of citrus fibers decrease continuously. Another possibility is that BM destroyed the cellular structure of citrus fibers and released small molecule polysaccharides, thus significantly reducing D_50_ of citrus fibers [37]. Consistent with our findings, BM treatment resulted in reduced particle sizes of potato, taro, and yam peels, and persimmon by-products [37]. According to [38], with the increase in grinding time, D_50_ of onion peel powder also decreased, but as the grinding time reached 18 h, D_50_ value did not decrease further.

### 3.3. Analysis of WHC

**Figure 2:**
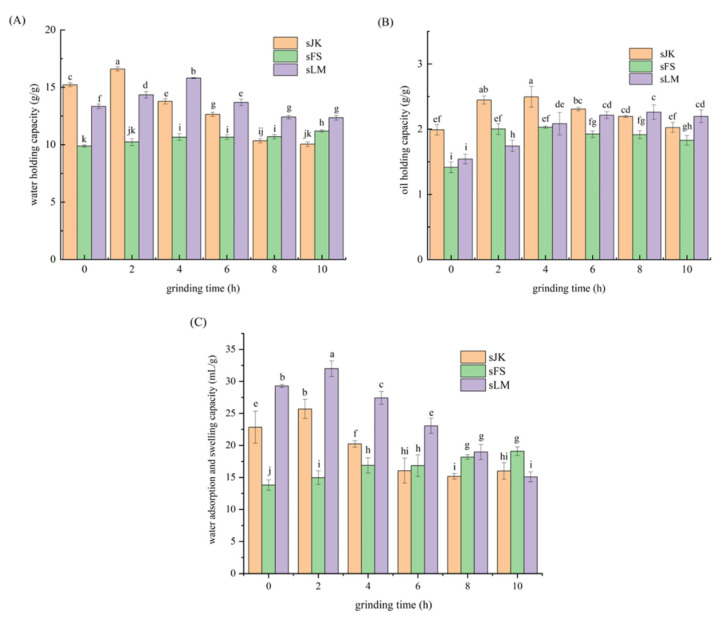
Effect of BM on WHC (**A**), OHC (**B**), and WSC (**C**) of three citrus fibers (JK, FS, and LM). Error bars represent the standard deviation of the mean of triplicate experiments. Different letters (a–k) indicate significant differences (*p* < 0.05).

Effects of BM on WHC of three citrus fibers are shown in Figure 2A. WHC of three citrus fibers was 15.21 ± 0.17 g/g, 9.88 ± 0.08 g/g, and 13.33 ± 0.21 g/g. After BM, WHC of citrus fibers JK and LM initially increased and then decreased. The reason for this may be that interfacial tension was reduced by BM and water binding sites were exposed to water [39]. During the BM process, the particle size of citrus fiber was reduced, and hydrophilic groups in the insoluble cellulose were exposed, thus resulting in the increase in WHC of LM and JK. The authors of [40] found that WHC of fibers was related to the porous structure formed by polysaccharide chains, which held a large amount of water through hydrogen bonds; however, it could be damaged with longer milling times, thus reducing WHC of JK and LM. The authors of [41] also found that WHC of asparagus leaf by-product powder decreased gradually from 6.2% to 3.8% with increasing grinding time from 0 to 6 h (*p* < 0.05).

However, WHC of FS increased significantly with the increase in grinding time. Potentially, WHC of dietary fiber was positively correlated with the content of SDF [42]. Among the three citrus fibers, FS had the highest SDF content, and it gradually increased with the extension of grinding time, so WHC of FS also showed an increasing trend. Specifically, WHC of citrus fibers LM, JK, and FS was increased by 18.52%, 9.07%, and 13.15% (*p* < 0.05), respectively, after being crushed by BM for 2, 4, and 10 h. LM and JK have more IDF (shown in Table 1).

### 3.4. Analysis of OHC

Citrus fiber has a large number of lipophilic groups, and the spatial structure is a loose network structure, which can absorb and bind a certain amount of oil, so it has OHC. Effects of BM on oil holding properties of three citrus fibers are shown in Figure 2B.

The authors of [23] showed that micronization improved OHC in Fraction 1 (>2 mm) of olive pomace, which was separated in a 2 mm sieve, but that of its Fraction 2 (<2 mm) was slightly declined (*p* < 0.05). The authors of [43] found that there were no significant differences in OHC of four citrus fibers, indicating that homogenization had little effect on OHC. The authors of [3] modified citrus fibers by water media BM; the results showed that this modification method could further enhance OHC of citrus fibers.

**Figure 3:**
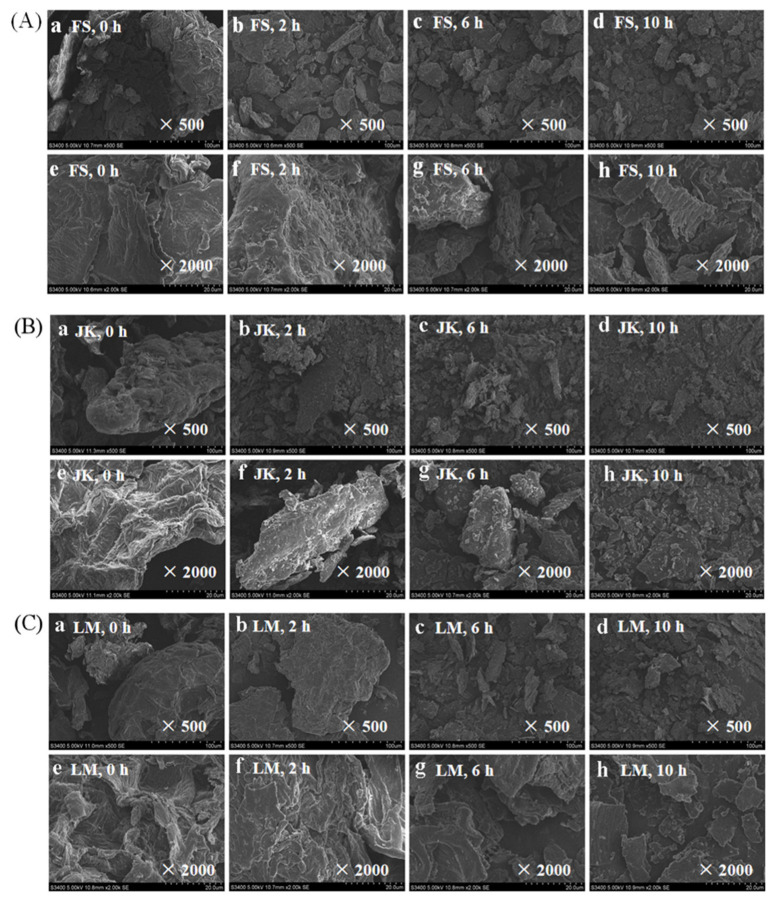
Effect of BM on the apparent structure of three citrus fibers ((**A**) was FS, (**B**) was JK, and (**C**) was LM). The magnifications of a–d (0, 2, 6, and 10 h) and e–h (0, 2, 6, and 10 h) were 500× and 2000×, respectively.

In our study, OHC of three citrus fibers LM, JK and FS was significantly improved during BM (*p* < 0.05). Compared with the untreated sample, OHC of LM and JK after BM for 4h was increased by 25.1% and 46.1%, and OHC of FS after BM for 8h increased by 41.8% (*p* < 0.05). This is because the spatial structure of three citrus fibers was looser after BM, and more lipophilic groups were released. The morphology analysis (shown in Figure 3) was consistent with the change in OHC, indicating that a coarse surface, loose structure, and particles with good dispersion were more favorable to retain oils in citrus fibers [3]. However, after prolonged BM treatment, the network spatial structure of JK and LM was destroyed, so it could not absorb more oil. Therefore, OHC of LM and JK showed a trend of rising first and then falling.

### 3.5. Analysis of WSC

Citrus fiber has WSC, probably due to its loose network space structure with hydrophilic groups, which can absorb a certain amount of water [44]. The effects of BM on WSC of three citrus fibers are shown in Figure 2C. WSC and WHC of three citrus fibers showed the same change trend.

Compared with the unmodified samples, WSC of citrus fibers LM and JK increased by 9.28% and 12.45%, respectively, after 2 h of BM (*p* < 0.05). After milling for 10 h, WSC of citrus fiber FS was increased by 38.3% (*p* < 0.05). Furthermore, the porosity and specific surface area of LM and JK were increased after BM for 2 h, and more hydrophilic groups were exposed [16], so its WSC could be improved. However, after BM for 4–10 h, the side chain structure and spatial structure of the cellulose were destroyed, leading to the decrease in WSC of LM and JK. In addition, WSC of FS that contained high water-soluble dietary fiber increased with the increase in BM time, due to the fact that its hydrophilic groups in water-soluble dietary fiber were exposed. More polar groups and water binding sites of citrus fiber were exposed after BM, resulting in an increase in WSC of FS [45]. The rice bran was crushed by a grater and then the coarse powder was obtained through an 80-mesh sieve. The fine powders and superfine powders were further obtained by regulating the grinding time. The result proved that WSC of superfine powders from rice bran dietary fiber was obviously higher than that of its coarse powders and fine powders [18]. Chitrakar et al. also found that WSC of dietary fiber powders from asparagus leaves increased from 25.74% to 32.47% with the increase in grinding time [41]. This is because the porous matrix structure of cell wall polysaccharides was damaged by BM, which enables them to retain water through hydrogen bonds [46]. This is similar to the mechanism of increased WHC of citrus fibers.

### 3.6. Analysis of CAC

**Figure 4:**
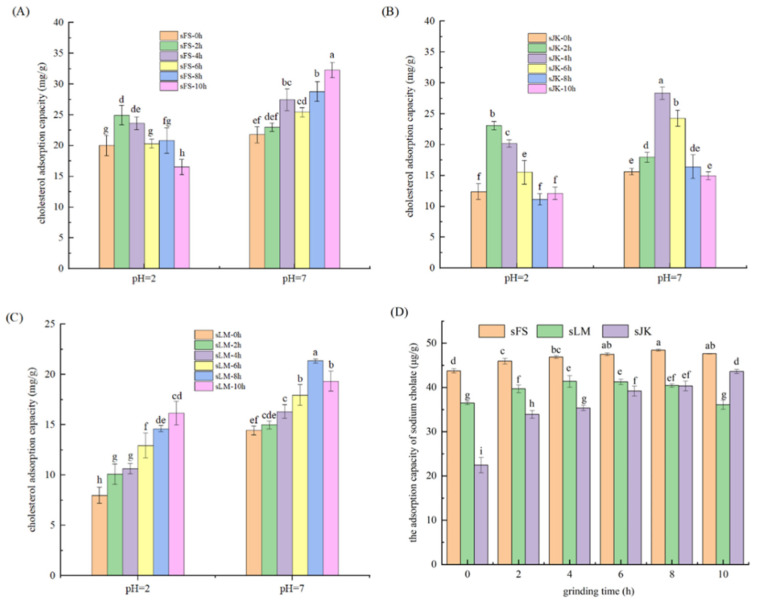
Effect of BM on cholesterol adsorption capacity (**A**–**C**) and the adsorption capacity of sodium fibrous cholate (**D**) of three citrus fibers (JK, LM, and FS). Error bars represent the standard deviation of the mean of triplicate experiments. Different letters (a–i) indicate significant difference (*p* < 0.05).

The influence of BM on CAC of three citrus fibers FS, JK, and LM is shown in Figure 4A–C. CAC can be associated with porosity and available surface area of dietary fibers [47].

After BM, CAC of three citrus fibers was significantly increased at pH 7.0 and pH 2.0 (*p* < 0.05). Among them, CAC of citrus fiber FS was the strongest as FS had more SDF. The internal structure of SDF had many pores and exhibited a strong adsorption capacity for cholesterol. Pectin and other SDF can improve the viscosity of foods in the intestinal tract, which can further absorb a large amount of cholesterol [48]. Compared with the unmodified samples, CAC of FS and LM at pH 7.0 increased by 48.34% and 47.95%, respectively, after 10 h of BM. CAC of JK increased by 41.50% after 6 h of BM. Because of the loose structure of citrus fibers, there were many holes on the surface after BM, which probably provided more binding sites for cholesterol [3].

In addition, CAC of three citrus fibers treated with pH 2.0 was lower than those treated with pH 7.0. Under acidic conditions, citrus fiber and cholesterol had partial positive charges and had a repulsive force, leading to the weakening of the binding force between citrus fiber and cholesterol [3], thus reducing CAC.

### 3.7. Analysis of Adsorption Capacity of Sodium Cholate

The influence of BM on the sodium cholate adsorption capacity of three citrus fibers is shown in Figure 4D. The adsorption capacity of sodium cholate of citrus fiber FS was the strongest, followed by that of LM and JK. After BM, the adsorption capacity of sodium cholate of three citrus fibers increased significantly (*p* < 0.05). Compared with the unmodified samples, the sodium cholate adsorption capacity of FS and JK increased by 10.74% and 48.64% after BM for 10 h; and that of LM increased by 13.07% (*p* < 0.05) after milling for 8 h. This was potentially due to the increase in SDF contents of three citrus fibers by BM, and the release of some chemical groups that adsorbed sodium cholate, which improved adsorption capacity of sodium cholate. Dietary fiber can effectively absorb sodium cholate, thus reducing the concentration of bile acid in the intestinal tract. As a result, the absorption of cholesterol was accelerated, and the blood glucose was then reduced in the body [49].

### 3.8. The Apparent Structure

The effects of BM (0–10 h) on the apparent structure of FS, JK, and LM are shown in Figure 3A–C, respectively. It can be observed at 500× magnification that, before, BM, LM, and JK had a spherical structure, whereas FS had a flake structure. After BM for 2 h, JK and LM changed from a spherical to a fragmented conformation. The flake structures of three citrus fibers became smaller and more uniform in the grinding time between 2 and 10 h. The authors of [41] also studied the effect of low-temperature BM on microstructural characteristics of asparagus leaf by-product. SEM demonstrated that the particles were irregular and broken form large particles to small particles in the process of BM.

It was observed at 2000× magnification that, before BM, compared with FS and JK, the overall structure of LM was looser and had more pores. In addition, the surface of JK and LM was looser, and the number of pores increased after BM for 2 h, which exposed more hydrophilic and lipophilic groups in citrus fiber [41], leading to improve its functional properties such as WHC and OHC. The surface of JK and LM was smooth at BM 6–10 h, while the surface of FS showed more pores, so the functional properties of FS increased with the extension in the grinding time.

### 3.9. Analysis of FTIR

**Figure 5:**
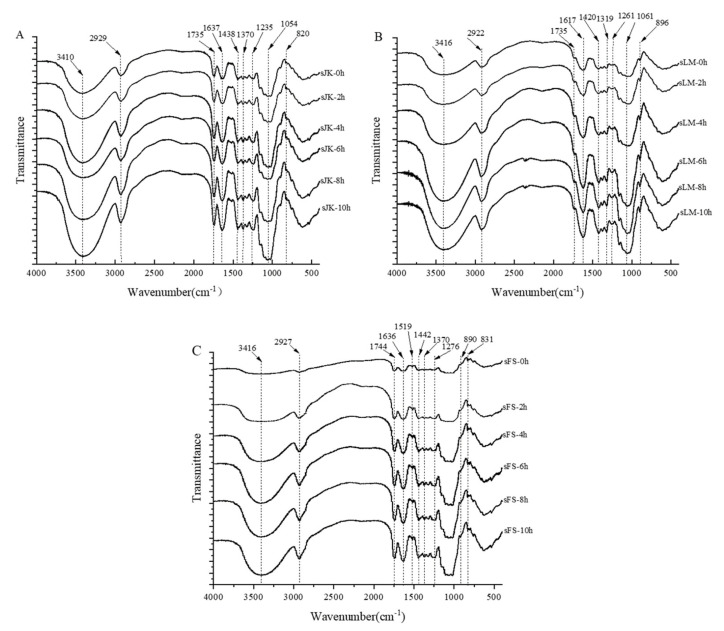
Effect of BM on infrared spectra of three citrus fibers ((**A**) was JK, (**B**) was LM and (**C**) was FS).

FTIR is an important indicator for evaluating proteins, fibers, and other substances [50,51]. Figure 5A–C respectively represent the effects of BM on FTIR of three citrus fibers. FTIR results of three citrus fibers were similar, but the curve transmittance results of the three citrus fiber samples were different. The peak vibration intensity of the three citrus fibers increased after BM due to the increase in the exposed -OH content in citrus fibers by BM [44]. As BM increased from 0 to 10 h, the peak values of three citrus fibers were enhanced in 2927 cm^−1^ bands (stretching vibration of C-H on the polysaccharide methylene), 1735 cm^−1^ and 1636 cm^−1^ bands (vibration of acetyl ester and uronic acid groups on the hemicellulose), and 1370 cm^-1^ bands (bending vibration of C-H in cellulose). The results showed that BM can loosen the structure of citrus fibers, and more functional groups were exposed. In addition, the characteristic bending or tensile vibration peak of lignin at 1519 and 1276 cm^−1^ did not decrease after BM, meaning that lignin was not degraded by BM [52].

## 4. Conclusions

This study was the first to compare the effects of BM on structure, physicochemical, and functional properties of three citrus fibers (LM, JK, and FS) from different sources. The results showed that the particle size of three citrus fibers reduced significantly after BM, and more hydrophilic and lipophilic groups were exposed, improving WHC, OHC, and WSC of citrus fibers. In addition, the content of SDF of LM, JK, and FS increased, and the adsorption capacity of cholesterol and sodium cholate was improved. Finally, it can be concluded that FS treated by BM for 8 h has good functional and adsorption properties. BM is a green mechanical grinding technology with the advantages of environmental protection and low cost. It is an effective technology to improve the characteristics of citrus fibers, and can thus widen the application of citrus fibers in the food industry as natural functional ingredients.

## Figures and Tables

**Table 1:** Effects of BM on IDF, SDF, and TDF contents of three citrus fibers (g/100 g).

Sample	IDF	SDF	TDF	IDF: SDF
sFS-0h	46.42 ± 0.28 ^j^	30.82 ± 0.18 ^c^	77.24 ± 1.00 ^e^	1.51
sFS-2h	45.72 ± 0.49 ^jk^	30.91 ± 061^c^	76.64 ± 0.83 ^e^	1.48
sFS-4h	44.88 ± 0.73 ^k^	31.52 ± 0.37 ^bc^	76.40 ± 0.72 ^ef^	1.42
sFS-6h	43.31 ± 0.92 ^l^	31.93 ± 0.07 ^b^	75.25 ± 0.99 ^fg^	1.36
sFS-8h	42.35 ± 0.23 ^lm^	32.19 ± 0.34 ^ab^	74.55 ± 0.10 ^g^	1.31
sFS-10h	41.31 ± 0.45 ^m^	32.73 ± 0.61^a^	74.05 ± 0.16 ^g^	1.26
sJK-0h	65.28 ± 0.25 ^cd^	18.99 ± 0.11 ^j^	84.27 ± 0.33 ^c^	3.44
sJK-2h	62.86 ± 0.74 ^e^	20.76 ± 0.61 ^hi^	83.63 ± 0.72 ^cd^	3.03
sJK-4h	61.41 ± 1.13 ^f^	21.70 ± 1.4 ^gh^	83.10 ± 0.27 ^cd^	2.82
sJK-6h	59.32 ± 0.33 ^g^	23.63 ± 0.53 ^f^	82.95 ± 0.19 ^cd^	2.51
sJK-8h	57.78 ± 0.66 ^h^	25.09 ± 0.30 ^e^	82.87 ± 0.65 ^d^	2.30
sJK-10h	56.17 ± 1.28 ^i^	26.50 ± 0.38 ^d^	82.67 ± 1.28 ^d^	2.12
sLM-0h	70.48 ± 0.56 ^a^	17.97 ± 1.05 ^j^	88.46 ± 0.73 ^a^	3.92
sLM-2h	69.61 ± 0.51 ^a^	18.23 ± 0.97 ^j^	87.85 ± 1.30 ^a^	3.82
sLM-4h	67.35 ± 0.94 ^b^	20.08 ± 0.54 ^i^	87.43 ± 1.46 ^a^	3.35
sLM-6h	66.45 ± 0.76 ^bc^	20.92 ± 0.07 ^hi^	87.38 ± 0.68 ^b^	3.18
sLM-8h	65.94 ± 1.05 ^c^	21.04 ± 0.91 ^hi^	86.98 ± 0.13 ^b^	3.13
sLM-10h	64.35 ± 0.47 ^d^	22.41 ± 0.23 ^g^	86.53 ± 0.35 ^b^	2.87

Note: Values with the different superscript letters in the same column are significantly different (*p* < 0.05).

## Data Availability

Data are contained within the article.
